# Radon Measurement Standards and Calibration

**DOI:** 10.6028/jres.095.001

**Published:** 1990

**Authors:** J. M. R. Hutchinson, R. Collé

**Affiliations:** National Institute of Standards and Technology

## Preface

This issue of the Journal of Research of the National Institute of Standards and Technology resulted from a one-half day seminar devoted to descriptions of the current status of the metrology of radon in international laboratories specializing in that area. The seminar, held June 5, 1989, at the Physikalisch-Technische Bundesanstalt (PTB), Braunschweig, Federal Republic of Germany was co-sponsored by the Low-Level Measurement Techniques Group of the International Committee for Radionuclide Metrology (ICRM), the Commission of European Communities and the International Intercomparison and Intercalibration Program (IIIP), now part of the International Atomic Energy Agency (IAEA). The IIIP has spearheaded the international effort to standardize radon measurements. It has, in particular, conducted intercomparisons between four selected laboratories that perform radon calibrations on different instruments. These four laboratories, which presently serve as primary reference laboratories for separate geographical regions, are the Australian Radiation Laboratory, U.S. Department of Energy Environmental Measurements Laboratory, U.K. National Radiation Protection Board, and U.S. Department of Interior Bureau of Mines. In addition to these four laboratories, other contributors to this seminar included national standards laboratories—National Institute of Standards and Technology (NIST) and the Italian Committee for Research and Development of Nuclear and Alternative Energies (ENEA)—as well as four select laboratories that have studied important measurement techniques and calibration procedures.

The seminar contributors were asked to describe their calibration measurement methods and in particular, to carefully assess and itemize their measurement uncertainties. A subsequent review, held in a workshop on the following day, established measurement needs and how present accuracies meet these needs. Often the user requires measurement accuracies in the 15–30 percent range. The quality assurance (QA) laboratories, such as the four primary IIIP laboratories, should therefore have uncertainties approximately one third of this—say 5–10 percent. The metrology laboratories of the ICRM are required to establish national and international standards of radon for the QA laboratories. One expects from this that for the national metrology laboratories to make a significant contribution to the radioactivity metrology of radon gas, their uncertainties should be significantly below 5 percent—a reasonable objective would be on the order of 2–3 percent.

The described methods are basically ionization chamber and scintillation cell measurements of gaseous ^222^Rn samples and gamma-ray measurements of the ^222^Rn daughters. The common standard is the NIST ^226^Ra standard, so that the various calibration measurements are tests of the ability of the laboratories to transfer the same calibrations (NIST standard) to an unknown using different techniques. Generally speaking, the QA laboratories have reached their goal of accuracies of 5–10 percent. There are, however, discrepancies and inconsistencies in the intercomparisons at present which may be significant. These and other related topics are discussed in these proceedings.

We are grateful to Drs. Debertin and Schrader and their staff at the PTB for making their facilities available and for being exemplary hosts. We are also grateful to Drs. Robert Holub, Earl Knutson, and Wayne Lower for their efforts in preparing the format of the meeting and helping contact the participants.

